# Bones hold the key to DNA virus history and epidemiology

**DOI:** 10.1038/srep17226

**Published:** 2015-11-27

**Authors:** M. Toppinen, M. F. Perdomo, J. U. Palo, P. Simmonds, S. J. Lycett, M. Söderlund-Venermo, A. Sajantila, K. Hedman

**Affiliations:** 1 Department of Virology, University of Helsinki, Haartmaninkatu 3, 00290 Helsinki, Finland; 2 Department of Forensic Medicine, University of Helsinki, Kytösuontie 11, 00300 Helsinki, Finland; 3 Roslin Institute, University of Edinburgh, Infection and Immunity Division, Easter Bush Edinburgh EH25 9RG, UK; 4 Helsinki University Hospital, Haartmaninkatu 3, 00290 Helsinki, Finland

## Abstract

DNA in human skeletal remains represents an important historical source of host genomic information and potentially of infecting viruses. However, little is known about viral persistence in bone. We searched ca. 70-year-old long bones of putative Finnish casualties from World War II for parvovirus B19 (B19V) DNA, and found a remarkable prevalence of 45%. The viral sequences were exclusively of genotypes 2 (n = 41), which disappeared from circulation in 1970´s, or genotype 3 (n = 2), which has never been reported in Northern Europe. Based on mitochondrial and Y-chromosome profiling, the two individuals carrying B19V genotype 3 were likely from the Soviet Red Army. The most recent common ancestor for all genotypes was estimated at early 1800s. This work demonstrates the forms of B19V that circulated in the first half of the 20^th^ century and provides the first evidence of the suitability of bone for exploration of DNA viruses.

The advent of new genomic and sequencing technologies has uncovered in recent years a myriad of viral sequences that were previously unknown to coexist with humans. Understanding viral evolutionary dynamics may be crucial for better monitoring and predicting the epidemiological trajectory of clinically relevant viruses. Thus, tracing the footprints of viruses in human remains may reveal important clues on their distribution, adaptation and on the influence that they may have on us.

DNA is most likely to be preserved across time in bone yet little is known about viral persistence in this organ. In the present study we searched for viral DNA skeletal remains of putative Finnish soldiers that went missing in action during the Second World War (WWII) and whose bodies had been decaying in the boreal forest in current Russian territory ever since. The remains have over the past 17 years been searched for and repatriated upon discovery to Finland for DNA-based identification^1^.

As a proof of principle, we examined these bones for human parvovirus B19 (B19V), a highly prevalent DNA virus establishing lifelong tissue persistence. B19V DNA has been detected in a range of tissues and organs^2^,^3^,^4^,^5^,^6^,^7^,^8^,^9^,^10^,^11^,^12^ but as of yet there is no direct evidence of its persistence in bone. The virus, however, replicates in erythroid progenitor cells in the bone marrow^13^,^14^,^15^ and also has been found in mesenchymal stromal cells, which can differentiate into cartilage and bone^16^.

B19V has three genotypes that are differently distributed around the globe. Of these, the most extensively studied and the one responsible for most current clinical cases is genotype 1. In Northern Europe, both genotypes 1 and 2 have been encountered in soft tissues of elderly individuals^7^,^8^,^17^; yet a clear perspective on the endemic prevalence of each type across the years is lacking, as the time of primary infection is not known.

The present work not only explores the suitability of bone for search of viral DNA but also reveals unambiguously the forms of B19V that circulated in the first half of the 20^th^ century.

## Results

### B19V DNA in bone

The B19V genomic prevalence was determined in DNA extracts of long bones of 106 anonymous World War II casualties. For this purpose, two quantitative PCRs (qPCR), Pan-B19V qPCR and VP-qPCR, targeting distinct conserved areas of the viral genome (non-structural [NS] and viral protein [VP] respectively), were used.

**Figure 1:**
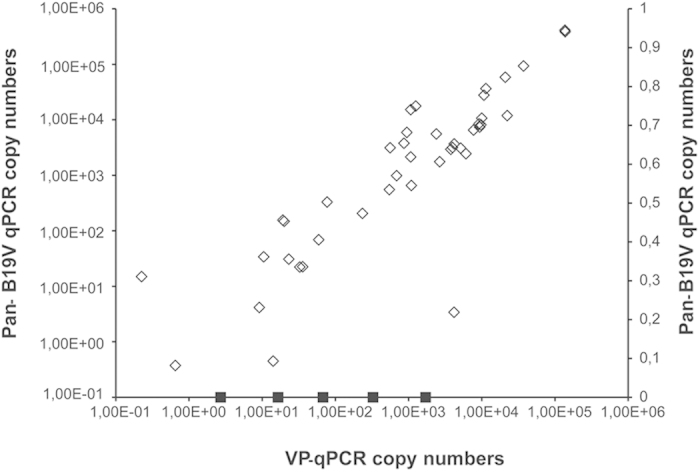
Correlation of B19V DNA copy numbers determined by Pan-B19V and VP qPCRs. Genomic B19V DNA was quantified by means of two in-house quantitative PCRs targeting distinct conserved regions of the viral genome (NS and VP). Represented are in the y-axis the genomic copies/1 μg of total DNA of individual samples as determined by the Pan-B19V qPCR. In the x-axis, genomic copies/1 μg of total DNA of individual samples as determined by VP-qPCR. In squares are represented the copy numbers of the five samples that were positive by the VP-qPCR only.

B19V DNA was detected in 48 samples (45%), of which 43 were positive by both qPCRs and five by the VP-qPCR only. Viral loads calculated by the Pan-B19V qPCR ranged from 3.7 × 10^−1^ to 4.1 × 10^5^ copies/1 μg of total DNA (mean 2.7 × 10^4^) and by the VP-qPCR from 2.2 × 10^−1^ to 1.4 × 10^5^ copies/1 μg of total DNA (mean 1.9 × 10^4^) (Fig. 1). The viral loads of the five samples positive solely by the VP-qPCR ranged from 2.7 × 10^0^ to 1.7 × 10^3^/1 μg of total DNA, thus the difference was not due to copy number or a lack of Pan-B19V qPCR sensitivity. The product of the VP-qPCR is shorter (121 bp vs 154 bp) and may therefore amplify more effectively the possibly fragmented DNA sequences in these bone samples.

All B19V qPCR products were sequenced. Among the samples positive with both qPCRs (n = 43), 41 showed 96.4%–99.6% total nucleotide identity to the B19V genotype 2 reference sequence (GenBank accession number AJ717293), while the remaining two samples (#12 and #38) showed 98.0% and 97.8% identity to genotype 3 (GenBank accession number AY083234). The five samples positive with only VP-qPCR showed 93.7–99.2% identity to genotype 2 (AJ717293).

No sequences of genotype 1, the currently circulating virus, were detected.

### B19 Evolutionary history

**Figure 2:**
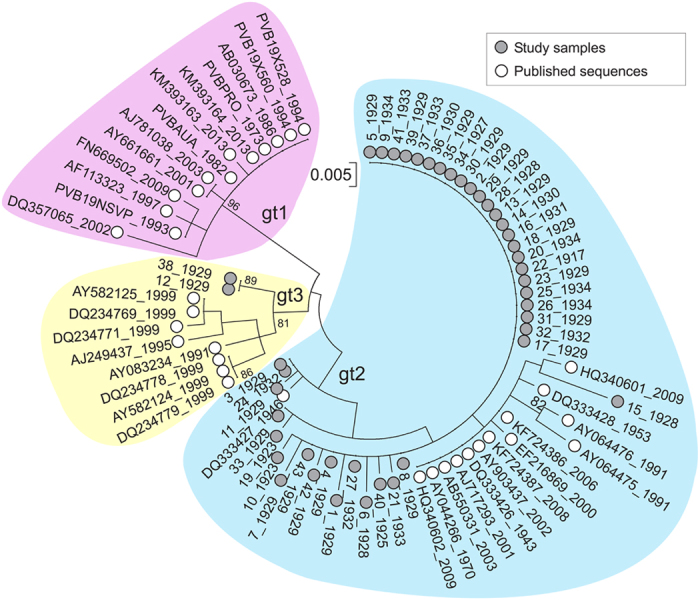
B19V phylogenetic tree. Maximum likelihood tree of the combined NS/VP region constructed using optimal substitution models (Kimura 2-parameter + invariant sites). Sample dates (acute infection; plasma) or predicted infection dates (assumed at 9 years of age; tissue) are shown on labels. Bootstrap re-sampling was used to determine robustness of groupings; values of 70% or greater shown.

**Figure 3:**
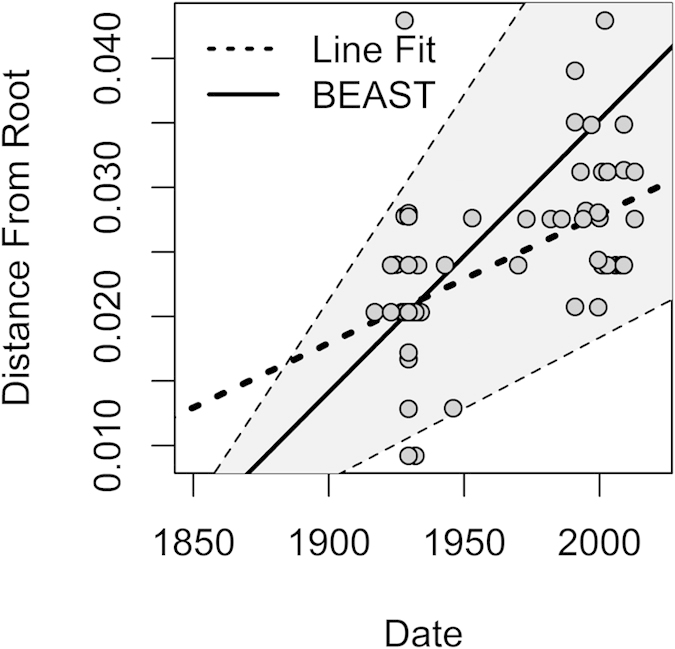
Genetic diversity over time. The genetic distance from the putative ancestral root from the maximum likelihood tree (Fig. 2) for each sample is plotted with the corresponding sampling date (circles). Dotted line: line of best fit for these points corresponding to a simple substitution rate estimate of 1 × 10^–4^ substitutions/site/year. Solid line (mean) and shaded region (95% HPD): overall substitution rate estimate from BEAST analysis: 2.1–2.2 × 10^–4^ (1.1–3.2 × 10^–4^) substitutions/site/year.

Sequences of B19V amplified using Pan-B19V and VP-qPCRs (positions 1850–2001 and 2709–2835 [numbered using the prototype PVBPRO/M24682 sequences]) were concatenated, and analysed together with other publically available, dated sequences of B19V genotypes 1–3. Phylogenetic analyses using a best fit maximum likelihood model showed that almost all sequences derived from the WWII study group adopted deeper positions in the tree compared to those obtained from more recent sampling (Fig. 2; sampling at the time of acute infection or estimated infection times shown in sequence labels). Plotting the genetic diversity from the putative ancestral root sequence to the observed sequences indicates time-related sequence change in B19V over the observation period (Fig. 3). The relatively deep branching of sequences in the genotype-2 clade is consistent with the early infection dates in the study subjects (Fig. 2).

**Figure 4:**
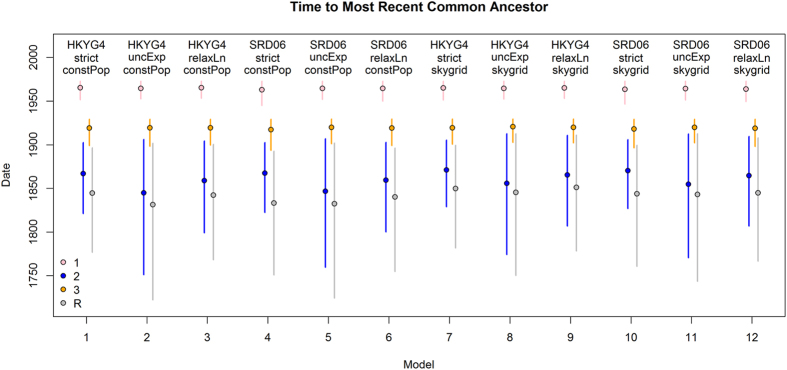
Time to most recent common ancestors of B19V genotypes. Estimated time to most recent common ancestor of genotypes 1 (pink), 2 (blue) and 3 (orange), as well as that of the whole tree (grey) using several models with BEAST. Models 1–3, and 7–9 additionally use the HKY nucleotide substitution model with site to site rate variation (HKYG4) and models 4–6 and 10–12 use the SRD06 codon partitioned substitution model. Models 1–6 also use a constant population coalescent model (‘constPop’), whereas models 7–12 use a Skygrid flexible effective population size prior model with 10 bins over 300 years (‘skygrid’). The model quoted in the main text is model 4, with the SRD06 substitution model, strict clock and constant population size.

Next, overall substitution rates and dates of origin for B19V variants were estimated using BEAST under a variety of substitution models, clock rate models and effective population size tree priors. All models gave similar results (See Fig. 4, and Supplementary Fig. S1 online), and the estimate from the best fit model was 2.11 × 10^−4^ substitutions/site/year with 95% Highest Posterior Density (HPD) intervals (1.18–3.16 × 10^−4^). The time to most recent common ancestor of all three genotypes was estimated as 1833 (95% HPD interval: 1751–1893) for the best fit model; and the TMRCA for genotypes 2 and 3 were 1868 (HPD: 1823–1902) and 1917 (HPD: 1893–1929), respectively (Table 1).

### Human DNA profiling

B19V genotype 3 has never before been encountered in Northern Europe. Thus, in order to gain insight into the geographical origin of the two individuals that were positive for this genotype, the mitochondrial (mt) and Y-chromosomal haplotypes were determined. For both samples, 631 bp hypervariable region profiles (HVR-1 and -2) were obtained. Likewise, full 23-locus Y-chromosome short tandem repeats profiles (Y-STR) were obtained for the sample 12, but for the sample 38 the loci DYS533, DYS643 and YGATAH4 could not be reliably typed. No matching mtDNA or Y haplotypes were found in the forensic in-house database consisting of 200 mtDNA and 893 Y-chromosomal Finnish haplotypes.

Sample 12 carried the mtDNA haplogroup H, with subhaplogroups H1e and H10a equally supported by HaploGrep^18^,^19^. The most probable Y-haplogroup for this sample is J1. Based on 9-locus “minimal haplotype”, the YHRD database^20^ suggested South-East European metapopulation as the most likely origin for this Y chromosome. There were 27 matching minimal haplotypes in the database distributed in all continents, but none in Northern Europe.

Sample 38 carried mtDNA haplogroup A, most likely subhaplogroup A12. The Y-chromosome most likely belongs to haplogroup N, and based on the Y-STR haplotype the YHRD database suggested Altaic origin. Only two matching minimal haplotypes were found in the database, one in the Yakut population in Siberia (Sakha Republic, NE Russia), and the other in Vilnius, Lithuania.

Of note, 20/41 B19V genotype 2 positive individuals were identified as Finnish, based on mtDNA comparison with living family members and detailed circumstantial information.

## Discussion

In the present study we provide evidence that viral nucleic acids can be detected in and genetically characterized from human bone. We found B19V DNA in 45% of long bones from casualties of the Second World War. Despite their long exposure (ca. 70 years) to varying environmental conditions in boreal wilderness^1^, the detection frequency was similar to that in modern soft-tissue biopsies^2^,^3^,^4^,^5^,^6^,^7^,^8^,^9^,^10^,^11^,^12^, demonstrating both good preservation of viral nucleic acids in human skeletal remains and high rate of viral DNA persistence *in vivo*. While the B19V genome persists in several tissue types its presence in bone has previously been only indirectly implied by Cantey *et al*.^21^ who reported radiological lucencies in a neonate with documented congenital B19V infection.

We found the viral sequences to be exclusively of genotypes 2 and 3. Our data with Finnish soldiers support the hypothesis^22^ that genotype-1 virus, the prototype and vastly predominant form in circulation throughout the world, emerged after WWII. Indeed, the present study for the first time sheds light on the epidemiological occurrence of B19V between 1923 and 1944, which correspond respectively to the median years of birth and death of the identified soldiers.

In order to calculate the rate of B19V sequence change, we used an assumption of primary infection at the age of 9 and no sequence change after infection as previously specified (Model A^22^). We obtained 2.1–2.2 × 10^−4^ substitutions/site/year (HPD interval 1.1–3.2 × 10^−4^) for the combined NS/VP region. This was lower than previous estimates based on the whole VP1 gene^22^ (3.6 × 10^−4^; HPD interval 2.5–4.6 × 10^−4^) although this latter estimate was based on a genome region showing substantially greater variability than the conserved NS and VP regions targeted in the current study for amplification. This nucleotide substitution rate is similar to that of other parvoviruses (e.g. porcine parvovirus (PPV) and canine parvovirus (CPV)) which have substitution rates approaching those of RNA viruses (10^−4^ substitution/site/year)^22^,^23^,^24^,^25^,^26^, despite using the cellular DNA polymerase for their replication. This favours a continuous evolution of important elements, such as the viral capsid, that have a profound role on virus-host interactions.

The HPD intervals of the most recent common ancestors (MRCAs) of genotype 2 and all three genotypes indicated that the genotype 2 infections in the study subjects occurred shortly after the original diversification of this genotype (~1868).

The detection of B19V genotype 3 in two individuals was surprising, as this type has never been reported in Finland and recorded to date in Ghana, Brazil and India^27^,^28^,^29^,^30^,^31^,^32^. We investigated the possibility that these subjects may not be of Finnish origin. Thus, we further studied their mtDNA and Y chromosome profiles in an attempt to clarify the geographical origin.

While the mtDNA haplogroup H in sample 12 is the most common in Europe (frequencies up to 50%), it also occurs in North Africa and the Middle East. The Y-STR data suggested a South-East European origin. The Y-haplogroup was probably J1, which is frequent in the Caucasus region and in the Middle East. In Finland this haplogroup is rare^33^.

For sample 38 the most probable mtDNA haplogroup was A, which is thus far unobserved in Finland yet found primarily in East and North Asia and among the indigenous peoples in America. The Y-STR data suggested haplogroup N that has a broad North Eurasian distribution. Although subhaplogroup N1c1 is very common in Finland, the particular haplotype found showed rather a Siberian (Yakut) than Finnish affinity. Thus, the combined mtDNA and Y chromosome data suggested rather Siberian origin for this individual.

While the determination of uniparental haplogroups based on mtDNA HVR-1 and -2 sequences or Y-STR profiles can rarely demonstrate geographical origins unambiguously, the combined data help to exclude Finnish origin for the two B19V genotype 3 positive individuals. It is likely that those remains belonged to soldiers of the Soviet Red Army conscripted from a much wider European and Asian range than other European combatants.

In the present work, we demonstrated that DNA viruses can be reliably detected and characterized in 7-decade-old human skeletal remains. Such material and potentially much older specimens may thus be suitable for investigations of epidemiologies and evolutionary histories of a range of other DNA viruses that might be similarly preserved. Further investigations are needed to clarify the suitability of this approach for RNA viruses, given the lability of RNA. Interestingly, influenza A virus RNA has been amplified in a biopsy of the lung of an influenza victim buried in permafrost since 1918^34^. Bones holding a clear history of an individual’s infectious encounters, may ultimately provide a much needed historical perspective on human viral diseases.

## Materials and Methods

### Bone samples

The bones had remained in the battlefields of Karelia district, near the border of Finland and Russia at the time of WWII. The climate in this region comprises four divergent seasons, with sub-zero temperatures and snow-cover during winter and long day lengths (≤20 hrs) during summer. The soil is generally acidic, with regional and micro-scale variations. The remains were initially considered Finnish in origin based on visual inspection of vestiges of military uniforms, ID tags or personal belongings^1^ under the coordination of the Association for Cherishing the Memory of the Dead of the War. DNA extracts of altogether 106 bone samples from WWII casualties were obtained from the Department of Forensic Medicine, University of Helsinki. The skeletal samples were obtained from femur (n = 79), humerus (n = 10), tibia (n = 5), radius (n = 4), fibula (n = 3), ulna (n = 2), calcaneus (n = 1), clavicula (n = 1) and os temporale (n = 1).

### Plasmid

A plasmid containing a near-full length B19V genotype 1 genome served in PCR as positive control and was utilized in dilution series as a standard to quantify the amount of B19V DNA in each sample.

### DNA purification

For each individual, a section of bone of approximately 10 cm was obtained for DNA analysis after anthropological investigation of the remains. In order to minimize the external DNA contamination and other impurities, bone samples were first cleaned mechanically using a toothbrush and distilled water, then shortly immersed in sodium hypochlorite (0.1%), and finally in ethanol (96.1 w-%). After these washing steps the bones were allowed to dry for 72 h in RT in a laminar hood.

The outermost surface was removed and bone powder for DNA extraction was obtained with a dentist drill (W&H Dentalwerk, Bürmoos, Austria) using sterilized blades. Approximately 1 g of fine bone powder was obtained and divided into two aliquots for extraction.

“Total lysis” DNA extraction^35^ proceeded with incubation of bone powder with 7.5 ml of lysis buffer (0.5 M EDTA, 1% N-lauroylsarcosine, pH 8.0) and 2 mg of proteinase K (Promega, USA) overnight at +56 °C with shaking. The unlysed debris was pelleted by centrifuging the sample for 10 min at 1300 × g and the supernatant (c. 7.5 ml) was transferred to Amicon^®^ Ultra-15 30 K tubes (EMD Millipore, USA) for concentration to a final volume of 500 μl. The supernatant was then washed once by adding 2 ml of distilled water in the lysate and centrifuging for 1 h at 2500 × g. After this, the extract underwent purification with QIAquick^®^ PCR Kit (Qiagen, Germany) according to manufacturer’s instructions to a final elution volume of 40 μl. Negative extraction controls (ddH_2_O) were run in parallel with the actual samples.

The amount of total DNA in each sample was measured by NanoDrop 1000 spectrophotometer (Thermo Scientific, USA).

### B19V quantitative PCRs

The PCR reaction mixes were produced in a clean room dedicated only to this purpose and the template was added in a separate room in a laminar hood dedicated to PCR work. Negative PCR controls were included in all assays. The laminar hoods were cleaned with Barrycidal 36 disinfectant (Fisher Scientific, United Kingdom) and 70% EtOH and were treated with UV always before use. Filtered tips and single-use disposable materials were utilized in each step. All the amplifications were done with the Stratagene Mx3005P (Agilent Technologies, USA) qPCR thermal cycler.

Two B19V qPCRs were used. The Pan-B19V qPCR targeting a 154 bp region of B19V NS1 gene was performed as described^36^.

The other B19V-qPCR was designed to amplify a 121 bp region of the VP gene using a forward primer (5′-CATGCCTTATCAYCCAGTARCAGT-3′) and a reverse primer (5′-AGGCCCAACATAGTTAGTACCG). The qPCR reaction consisted of 1 × Maxima SYBR Green Master mix (Thermo Scientific, Lithuania) with 0.03 μM of ROX passive reference dye, 0.6 μM each of the forward and reverse primers, 200 ng or 5 μl template DNA, and nuclease-free water to a final volume of 25 μl. After an initial denaturation at 95 °C for 10 min, the qPCR cycles were 95 °C for 15 sec and 58 °C for 1 min, altogether 40 cycles. After the amplifications, melting curve analysis was performed as follows: denaturation at 95 °C for 1 min, 45 °C for 30 sec, 0.01 °C/sec ramping from 45 °C to 95 °C, and finally 95 °C for 30 sec. The adaptive fluorescence baseline was automatically calculated by the MxPro–Mx3005P v. 4.10 software (Agilent Technologies) and the threshold for the quantification cycle (Cq) was set at 10 times the standard deviation of the mean fluorescence baseline in cycles 5–9.

The analytical sensitivity of the VP-qPCR assay was determined with serial dilutions of B19V genotype 1–3 plasmids at 500, 200, 100, 50, 20, 10 and 5 copies/reaction. After optimization, the VP-qPCR amplified and quantified all three B19V genotypes equally, with detection sensitivities of ≤10 copies/reaction of genotype 1–3 plasmid templates.

The analytical sensitivity of the Pan-B19V qPCR was ≤10 copies/reaction of genotype 1–3 plasmid templates, as previously specified^36^.

In order to analyse the specificity and functionality of the VP-qPCR, human DNA extracted from A549 cells (total of 0.5 μg) was used as template, by itself or spiked to 1.00E+02 copies/μl of B19V plasmids. The qPCR was unaffected by the human A549 DNA.

### B19V sequencing and phylogenetic analysis

The products of both qPCRs were purified with Diffinity RapidTip2 (Sigma Aldrich, USA) and sequenced with the corresponding qPCR primers at the Haartman Institute Sequencing Unit.

Phylogenetic analysis was performed on altogether 78 genotype-1, -2 and -3 sequences (of which 43 from this study) by constructing maximum likelihood trees using the optimal nucleotide substitution model identified in ModelTest (Kimura 2 parameter with invariant sites) as implemented in the MEGA6 package^37^. Genetic diversity for each of the sequences from the putative ancestral root sequence was calculated using Path-o-gen v. 1.4 (http://tree.bio.ed.ac.uk/software/pathogen/) and plotted together with a simple estimate of the overall substitution rate from the line of best fit (linear regression).

Dated sequences were further analysed using the BEAST package (v.1.8.1)^38^. In order to estimate the overall substitution rate (clock rate) and time to most recent common ancestor (TMRCA) the Shapiro-Rambaut-Drummond-2006 (SRD06) substitution model, strict clock model, and constant population size tree priors were used. Selections of other models (Hasegawa, Kishino and Yano [HKY] or General Time Reversible [GTR] substitution models; uncorrelated relaxed log normal clock or uncorrelated relaxed exponential clock; exponential growth, Bayesian skyline and Bayesian skygrid tree priors) were also considered and these produced comparable estimates for the overall substitution rate and MRCAs. The TMRCA and clock rate estimates for HKY/SRD06, strict/relaxed clock, constant population/skygrid can be found in Fig. 4 and Supplementary Fig. S1 (online). Comparison of these models by Bayes Factor (BF) test in TRACER (version 1.6, http://beast.bio.ed.ac.uk/Tracer) using Bayes Factor tests (Akaike Information Criteration^39^) revealed that the SRD06 model was preferred over HKY and GTR models (BF > 3), and that the more complex relaxed clock and variable population size models were not significantly better than the strict clock and constant population size models. Marginal likelihood estimation by stepping stone sampling and path sampling^39^, to more accurately distinguish between clock models, also confirmed that using the strict clock was most appropriate (difference in Log marginal likelihood <3). Hence final model selections were SRD06, strict clock, constant population size, and the final parameter estimates were drawn from the combination of two independent Markov Chain Monte Carlo (MCMC) runs consisting of 10 million steps after allowing 1 million steps burn-in (10%) per run.

### mtDNA and Y chromosome studies

Mitochondrial hypervariable segments 1 and 2 (positions 16024–16385 and 72–340) were sequenced as described previously^40^. The sequencing products were resolved using ABI3500XL sequencer and the data analysed using Sequencher v.4.8 software (Gene Codes). Based on the sequence data the haplogroup estimation was performed using HaploGrep based on Phylotree v. 16 (http://www.haplogrep.uibk.ac.at)^18^,^19^. The haplotype frequencies in different parts of the World were searched in the EMPOP database (version 23, release 11) including both the forensic and literature databases.

The Y-chromosome 23-locus Y-STR profiles were obtained using the forensic PowerPlex Y23 genotyping kit (Promega) using 1 μl (approximately 0.5–1 ng) of the DNA extract as a template. The PCRs were performed according to the manufacturer’s recommendations except that the total reaction volume was reduced to 12.5 μl (0.5× the recommended). The products were resolved using ABI3500XL sequencer and the data analysed using GeneMapper ID-X (Applied Biosystems, USA).

The Y-chromosomal haplogroups were estimated based on the Y-STR profiles using the web based Haplogroup Predictor (Whit Athey; http://www.hprg.com/hapest5/). The geographical origin of the haplotypes was assessed through database searches against the forensic YHRD database (Sascha Willuweit & Lutz Roewer, release R48, 10^th^ November 2014; http://yhrd.org).

## Additional Information

**How to cite this article**: Toppinen, M. *et al*. Bones hold the key to DNA virus history and epidemiology. *Sci. Rep*. **5**, 17226; doi: 10.1038/srep17226 (2015).

## Supplementary Material

Supplementary Information

## Figures and Tables

**Table 1:** Dates of most recent common ancestors of B19V genotypes.

Sequence Group	No. Sequences	Date Range	Date of MRCA*
All	78	1917–2013	1833 (1751–1893)
Type 1	13	1973–2013	1963 (1945–1972)
Type 2	55	1917–2009	1868 (1823–1902)
Type 3	10	1929–1999	1917 (1893–1929)

MRCA, most recent common ancestor

^*^Mean value and highest posterior density interval
