# Supplementary material for: Bones hold the key to DNA virus history and epidemiology
Source: Sci Rep. 2015 Nov 27;5:17226. doi: 10.1038/srep17226 (PMC4661702; doi:10.1038/srep17226)
Supplement: Supplementary Information [file srep17226-s1.pdf]

Supplementary Figure

Bones hold the key to DNA virus history and epidemiology

Toppinen M[a,1], Perdomo MF[a,1], Palo JU[b], Simmonds P[c], Lycett SJ[c], Söderlund-Venermo M[a], Sajantila A[b] and Hedman K[a,d,*].

[a]Department of Virology, University of Helsinki, Haartmaninkatu 3, 00290 Helsinki, Finland.

[b]Department of Forensic Medicine, University of Helsinki, Kytösuontie 11, 00300 Helsinki, Finland.

[c]Roslin Institute, University of Edinburgh, Infection and Immunity Division, Easter Bush Edinburgh EH25 9RG, UK.

[d]Helsinki University Hospital, Haartmaninkatu 3, 00290 Helsinki, Finland.

[1]Equal contributions

*corresponding author, Email: klaus.hedman@helsinki.fi

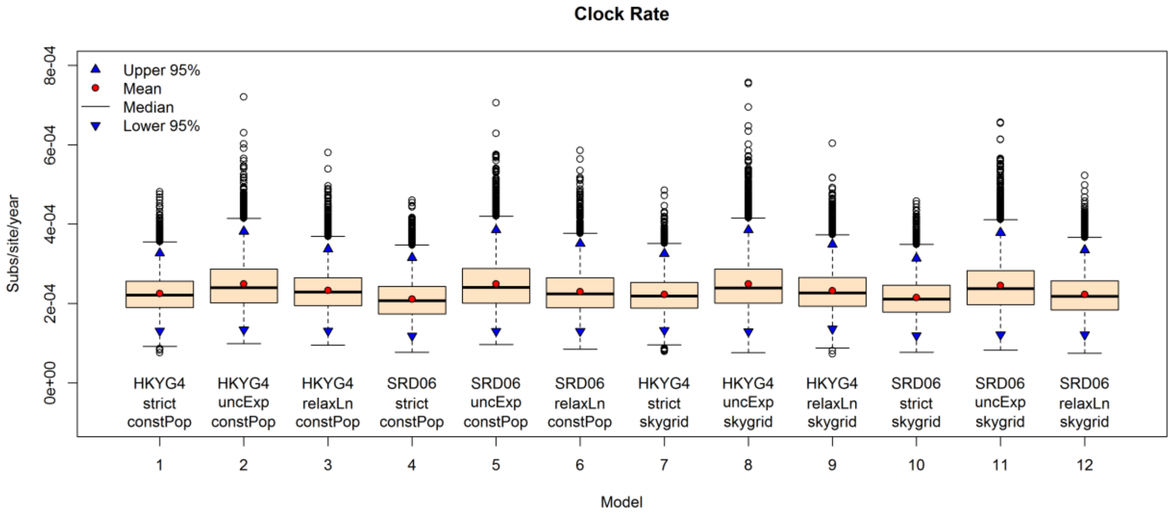

Supplementary Fig. S1. **Overall clock rate estimates for Genotype 1, 2 & 3 using several models with BEAST**. Clock models tested were the strict clock ('strict', models 1, 4, 7, 10); uncorrelated exponential clock ('uncExp', models 2, 6, 8, 11); and uncorrelated relaxed lognormal clock ('relaxLn', models 3, 6, 9, 12). Models 1-3, and 7-9 additionally use the HKY nucleotide substitution model with site to site rate variation (HKYG4) and models 4-6 and 10-12 use the SRD06 codon partitioned substitution model. Models 1-6 also use a constant population coalescent model ('constPop'), whereas models 7-12 use a Skygrid flexible effective population size prior model with 10 bins over 300 years ('skygrid'). The model quoted in the main text is model 4, with the SRD06 substitution model, strict clock and constant population size.
